# Internal Wave Turbulence Near a Texel Beach

**DOI:** 10.1371/journal.pone.0032535

**Published:** 2012-03-05

**Authors:** Hans van Haren, Louis Gostiaux, Martin Laan, Martijn van Haren, Eva van Haren, Loes J. A. Gerringa

**Affiliations:** 1 Royal Netherlands Institute for Sea Research, Den Burg, The Netherlands; 2 CNRS/Grenoble-INP/UJF-Grenoble 1, Laboratoire des Ecoulements Géophysiques et Industriels, Grenoble, France; 3 OSG de Hogeberg, Den Burg, The Netherlands; Plymouth University, United Kingdom

## Abstract

A summer bather entering a calm sea from the beach may sense alternating warm and cold water. This can be felt when moving forward into the sea (‘vertically homogeneous’ and ‘horizontally different’), but also when standing still between one’s feet and body (‘vertically different’). On a calm summer-day, an array of high-precision sensors has measured fast temperature-changes up to 1°C near a Texel-island (NL) beach. The measurements show that sensed variations are in fact internal waves, fronts and turbulence, supported in part by vertical stable stratification in density (temperature). Such motions are common in the deep ocean, but generally not in shallow seas where turbulent mixing is expected strong enough to homogenize. The internal beach-waves have amplitudes ten-times larger than those of the small surface wind waves. Quantifying their turbulent mixing gives diffusivity estimates of 10^−4^–10^−3^ m^2^ s^−1^, which are larger than found in open-ocean but smaller than wave breaking above deep sloping topography.

## Introduction

The present oceanographic view is that in shallow waters of only a metre deep, temperature (T) must be homogeneous by turbulent tidal and wind mixing. In contrast, when asked, Italian mothers, lazily chatting in water up to their waist in summertime Adriatic Sea while overlooking their offspring, experience not a constant water temperature but slowly alternating warm and cooler blobs of varying height [Margiotta, pers. comm.., 2011]. Now, this is an area where surface water temperatures reach T_w_ = 29°C, near the range of neutral human ambient skin-temperature at which both warm and cold fibers are active. Human thermoception starts at 0.5–1°C T-difference, from physiology experiments of small body-surfaces around 32°C [1]–[3], but body cooling reduces local thermal sensitivity [4]. However, regular beach visitors in countries like the Netherlands (T_w_≤20°C) confirm: slow minute-like T-variations can be sensed, when seas are calm.

Here we investigate using high-resolution T-sensors whether bathers in the North Sea experience physical phenomena like ‘fronts’ and ‘internal waves’ and what temperature differences their bodies sense under relatively cold conditions. Such physical phenomena common in the ocean interior have never been quantified close to shore. The present study proofs suppositions about shoreward extent of internal waves from historic observations in ≥5 m water off Scripps and Oceanside piers in the Pacific [5], [6].

As seas (and oceans) are heated from above and warm water is less dense (“lighter”) than cold water, they are generally stably stratified in density. Fresh above salty water contributes also positively to density stratification. A stratified sea supports motions in its interior that basically go unnoticed at the sea surface: internal waves (IW) which propagate much slower but with much larger amplitudes than surface wind waves (SWW). Density stratification hampers vertical turbulent exchange of heat and nutrients due to its stability. Counteracting destabilization occurs internally, as IW may break, and even more energetically near lower and upper boundaries due to (tidal) current friction at the bottom and atmospheric cooling and wind shear stress at the surface. For the southern North Sea, winds, SWW-breakers and >0.4 m s^−1^ tidal currents are commonly thought to mix solar heating from surface to bottom year-round [7], except for fresh-water outflows.

We will show that IW can occur even at one of the windiest spots of the Netherlands, near a beach of Texel-island. This is where normally SWW-breakers reside, except after a prolonged period of fine days for the entire region with strong insolation that is not mixed away by atmospheric and tidal boundary stresses. We quantify the rate of turbulent mixing by these ‘beach-IW’.

## Materials and Methods

No specific permits were required for the described field studies. No specific permissions were required to perform NIOZ measurements at Texel beach. The location is not privately-owned or protected in any way. The field studies did not involve endangered or protected species.

**Figure 1:**
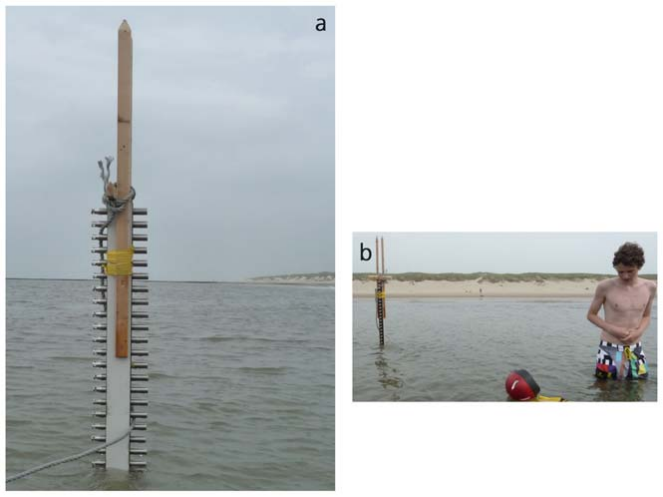
Instrument pole (upper sensor 1.60 m above the bottom) near Texel beach with shoreward small surface wave breakers (thin white line close to the beach). a. Along-beach view. b. Beach-view with differential water temperature sensing. The photos are taken an hour before low-water.

A total of 36 ‘NIOZ4’ self-contained temperature (T) sensors sampling at 2 Hz, with precision better than 0.001°C [8], were mounted at 0.042 m vertical intervals on a wooden pole (Fig. 1). The lowest sensor was 0.13 m from the bottom. Two sensors failed, including the lowest. The pole was fixed to the sandy, slightly undulating and weakly sloping bottom using two concrete slabs, weighing ∼40 kg each (in air). On six selected days of calm weather and small SWW it was moored at 53° 02.944′N, 04° 42.800′E (near Texel beach-pole 13), H = 0.6 m at low-water (LW), during periods varying from half an hour to nearly a tidal period within a span of 14 days in June/July 2010. At Texel beach we do not need specific permits for water temperature measurements. This time-span covered an atmospheric high-pressure system over the region and calm, warm weather. Distance to shore (high-water, HW, mark) was approximately 100 m. Predicted tidal range is 1.8±0.2 m, which includes spring-neap variation. Meteorological data are available from airport Den Helder (15 km southward). SWW amplitudes and periods are estimated from air-water surface passing the T-sensors.

The T-data are used as a conservative estimate for density (ρ) variations, using standard relation, δρ = −αδT, α = 0.23 kg m^−3^ °C^−1^ denoting the thermal expansion coefficient under local conditions. Lacking salinity (S) data it is assumed that only temperature contributes to δρ. As will be shown in Section 3, evaporation (salting the surface) contributes less to density than heating. Differential horizontal advection will reinforce solar heating stratification, because in summer the fresher inland tidal-flat Wadden Sea, bordering Texel on its eastern side, is warmer than the saltier North Sea [9]. Hence, the above density-temperature relationship is a conservative one.

Vertical turbulent eddy diffusivity K_z_ and turbulent kinetic energy dissipation rate ε are estimated by calculating ‘overturning’ scales using T-data. These scales are obtained after reordering every time-step potential density (temperature) profile, which may contain inversions, into a stable monotonic profile without inversions [10], [11]. After comparing raw and reordered profiles, displacements (d) are calculated necessary for generating the stable profile. A certain threshold applies to disregard apparent displacements associated with instrumental noise. This is very low for NIOZ-thermistor data, <5×10^−4^ °C [8]. Then,
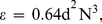
(1)where N denotes the buoyancy frequency computed from the reordered profile and the constant follows from empirically relating the overturning scale with the Ozmidov scale L_O_ = 0.8d [12]. Using K_z_ = ΓεN^−2^ and a mixing efficiency for conversion of kinetic into potential energy of Γ = 0.2 [13], we find,
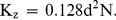
(2)


## Results

**Figure 2:**
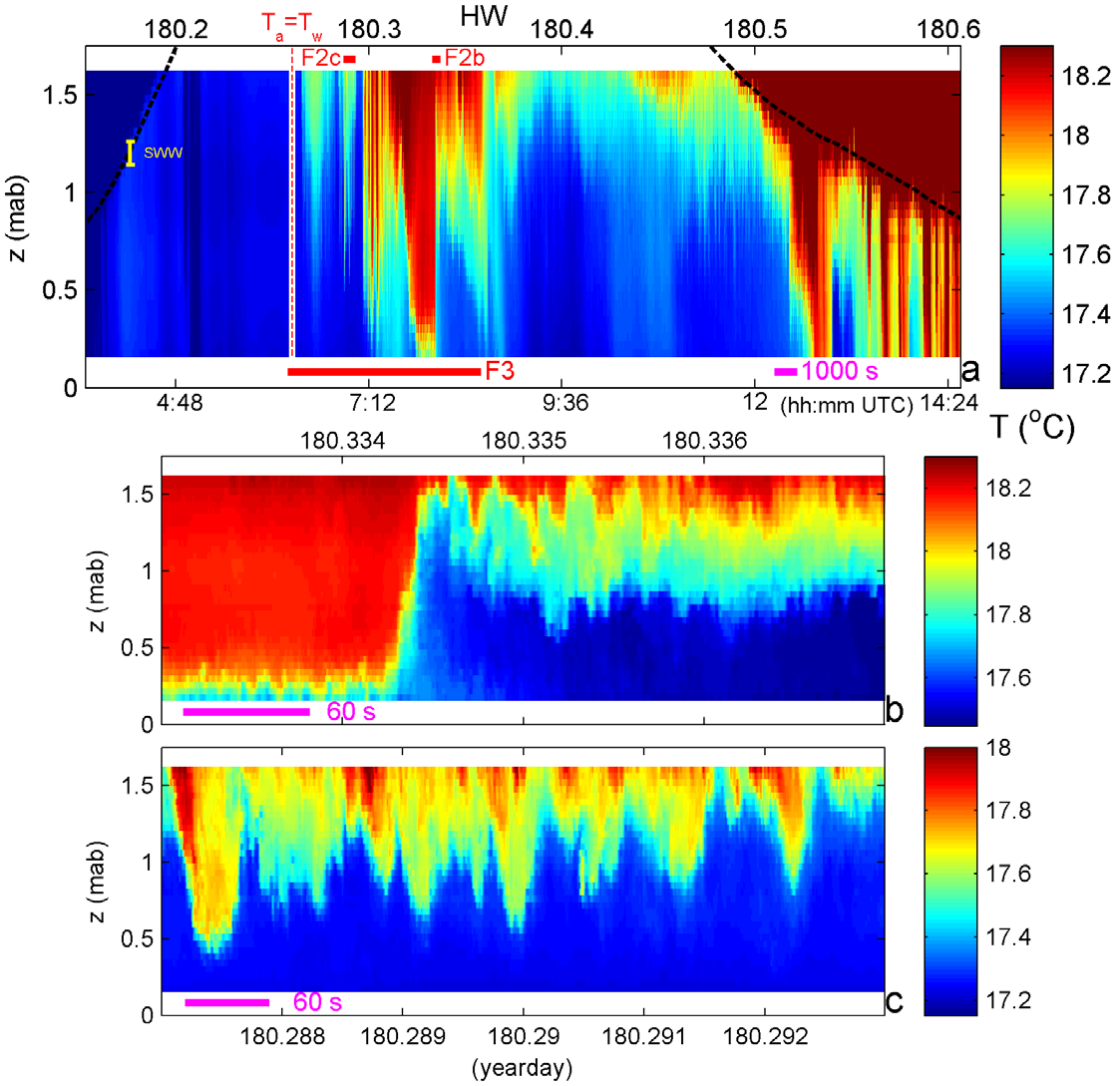
Almost one tidal period overview of detailed temperature (T) variations near Texel beach in early summer. The vertical scale is 1.75 m with reference to the bottom. For display purposes, the colour-scale does not completely cover the observed water temperature range of [17.05, 18.97]°C. a. The small red bars indicate zooms in b., c. and the large one indicates the period of Figure 3. The vertical red-in-white line indicates when T_a_ = T_w_ using upper thermistor in water and atmospheric data from Den Helder-airport. The black dashed curves indicate the predicted tidal sea-level height variation (sensors in air above it) and the yellow bar on the left spread of surface wind wave height, estimated from the water surface passing T-sensors. Time (UTC) is 0.33 hours before local SolarTime. b. Zoom of a front and trailing 80 s period IW and shorter period IW-turbulence motions. c. Zoom of high-frequency (60 s period) internal waves and shorter scale overturning.

During the fortnight of observations, water warmed steadily on average. The short-scale phenomena to be described here were observed in all six records, although with some variations in intensity. We analyze the longest observation period of 11 hours on 30 June 2010 (yearday 180). The pole was moored around sunrise, time of LW+1 h. Until day 180.26 (06:30 UTC; 08:30 LT) humidity was 100%, wind speed |W|<2 m s^−1^, air temperature T_a_<T_w_ and in sea no T-differences were observed (Fig. 2). Cloud-cover remained high (60–100%) the entire day, with some sunshine between days 180.3 and 180.4 when humidity reached a minimum of 80%. A daily evaporation sum of 0.7 mm is calculated for these conditions [14], implying a salting of 0.02 PSU m. To balance this density contribution one requires ∼0.1°C of warming; the observed temperature differences were much larger. Potentially stratification counteracting, fresh ground water leakage is found little important, as it would generate free convection in the overlying saltier sea water, hence complete homogenizing. T_a,max_ = 22°C around day 180.55, when the easterly wind-speed equaled 4–5 m s^−1^. Such winds could increase stratification in concert with lateral density gradients, but variations as presented below were also observed during periods of no wind. Easterly winds created only small surface waves near the pole, as the beach is exposed to the West. SWW-height including swell was about 0.1 m (Fig. 2a, yellow bar) with 3–10 s periods.

T-observations in the sea (Fig. 2a) varied between 17.05 and 18.97°C (in saturated to the right). In the beginning of deployment when T_a_<T_w_, stratification was very weak, although the upper layer was still 0.03°C warmer than near-bottom, and occasionally even, apparently unstable (cool above warm). As soon as T_a_>T_w_, well-stratified water passed T-sensors that were now all below surface (at HW sea level is about 0.90 m above the highest sensor). Around HW more than 1°C warmer water passed in different ways: slowly varying with periods of a few hours (Fig. 2a after day 180.265), with periods of half an hour (180.30–180.38) and more rapidly with changes every 60–90 s (Fig. 2b,c): trains of IW with vertical amplitudes varying between 0.2 and >1.0 m. T-variations thus occupied a substantial part of the water column. Such 0.5–1°C temperature differences were sensed by team members, regularly swimming or standing in the sea near the pole without disturbing the measurements (Fig. 1b).

Heating from the atmosphere does not directly force IW, as stable warm water initially spreads like a pancake over colder water. IW-amplitudes are much larger than SWW's and their periods O(100–1000 s) are much longer than 10 s.

Details show that irregular internal motions also occur that can have periods of 10–30 s (Fig. 2b,c). These cannot be interpreted as freely propagating IW, as local stratification has a smallest buoyancy period of T_N_≈60 s when calculated in extremely thin layers <0.1 m, even though using the conservative density-temperature relationship. Instead, these short-period motions represent turbulent overturns, e.g., day 180.3346 (Fig. 2b) and days 180.2876, 180.2912 (Fig. 2c), following different (IW-) currents above and below shearing an interface and rolling it up. These overturns have periodicities between IW and SWW [15]. Shear magnitude (|**S**|) is not measured here, but extrapolating from historic central North Sea observations [16], we can expect |**S**|∼N∼O(10^−1^ s^−1^).

A transition between warm and cold water passes, which is more than 1.2 m in height (Fig. 2b). It mimics a frontal passage, like in the atmosphere. Near the bottom it seems to be unstable (Fig. 2b, day 180.3345: water near the bottom warmer than halfway up) and may thus break thereby generating turbulence. The “waves” riding the near-bottom stratification are not propagating IW, but have SWW-amplitudes and periods: pumping up- and down the water column. The larger amplitude and -period front-trailing waves include asymmetric turbulence-like motions.

A train of finescale IW with turbulent mixing cores and overturning (Fig. 2c) shows every minute T-variation of 0.5°C across ∼0.5 m in the vertical.

**Figure 3:**
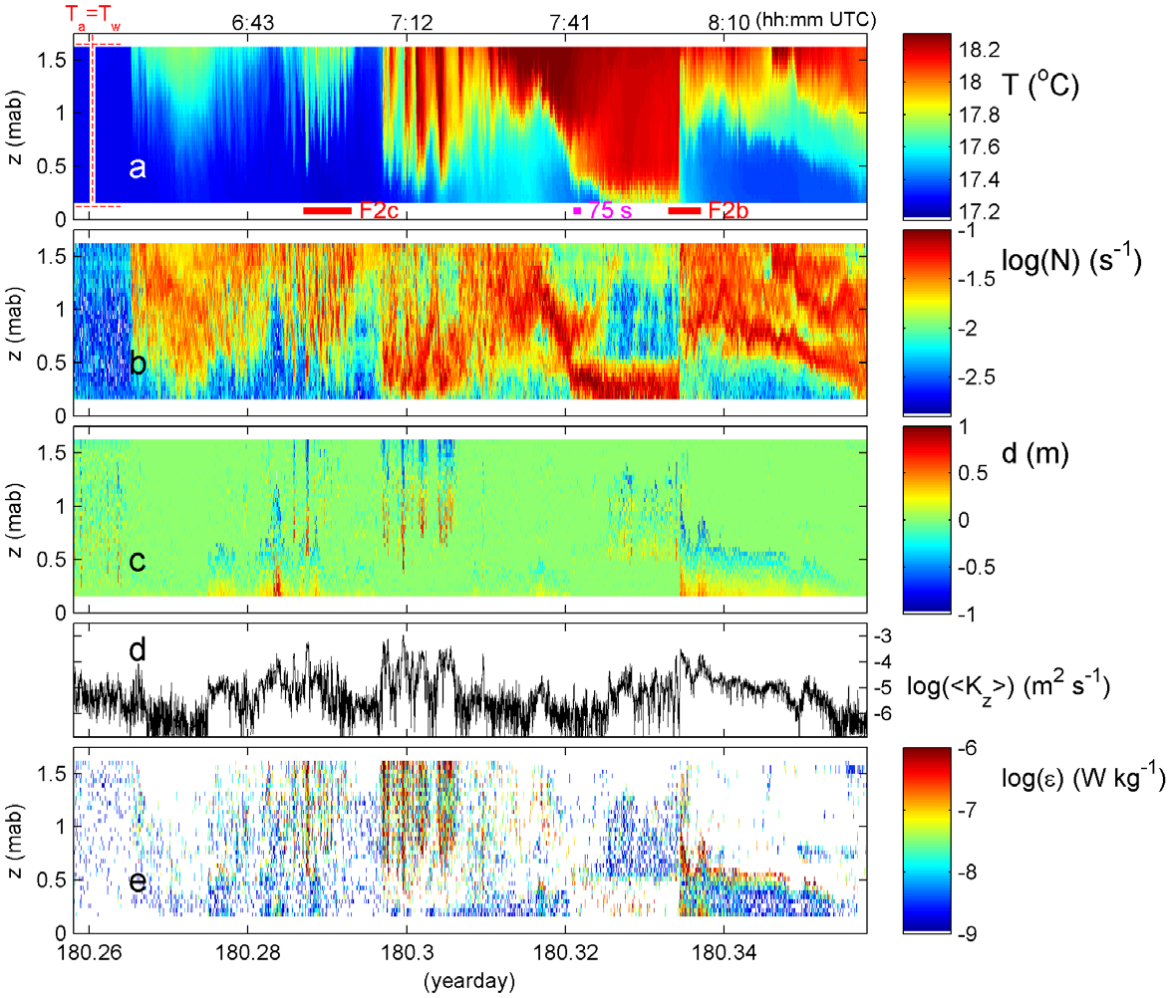
Depth-time series of T and computed turbulence parameters during 2.4 hours around high-water. a. Observed T-data. b. Stable stratification after reordering a. to stable profiles every time step and using δρ = −0.23δT kg m^−3^ °C^−1^ (see text). c. Overturning displacements following comparison of a. with its reordered data. d. Time series of vertically averaged eddy diffusivity using (2). e. Turbulence dissipation rate, estimated using (1). White also indicates values below threshold.

We quantified this turbulent mixing (Fig. 3). Until day 180.265 (Fig. 3a; red-in-white line indicates independent estimate T_w_ = T_a_) the near-homogeneous layer (Fig. 3a,b) is weakly stable with occasional overturns (Fig. 3c). This mixing is likely driven following nighttime air-sea interaction. It provides vertical average <K_z_> = 1–3×10^−5^ m^2^ s^−1^ (Fig. 3d). The associated heat flux (not shown) and turbulence dissipation rate (Fig. 3e) are not very large (ε<10^−8^ W kg^−1^) because stratification is so weak.

Transition (day>180.265; T_a_>T_w_) is abrupt from weak to strong stratification. After an overturning front, turbulence parameters drop one-two orders of magnitude, even though T-variation is initially a few tenths of degrees. However, periods when turbulence approaches molecular diffusion/noise level values (10^−7^≤K_z_≤10^−6^ m^2^ s^−1^; ε≤10^−9^ W kg^−1^) are rare and through the entire range small spots of <K_z_>≈10^−5^ m^2^ s^−1^; ε≈10^−8^ W kg^−1^ are observed. These spots are associated with short-period weak IW-overturning. IW-amplitudes, stratification and turbulence gradually intensify until day 180.31. Highest values (K_z_ = 10^−3^ m^2^ s^−1^; ε = 10^−5^ W kg^−1^) are in the upper half of the observation z-range.

After day 180.31, when larger-scale IW moves up and cooler bottom water move in, turbulence becomes weaker (in Fig. 3) until the large warming wave nearly reaches the bottom (day 180.325–180.333). At 0.3–0.4 m strong stratification is found (N_max_ = 10^−1.2±0.1^ s^−1^; (T_N_)_min_ = 75±15 s) below a warm (Fig. 3a) weakly stratified, turbulent core (Fig. 3b–e). The large, ∼half hour period wave contains multiple smaller, weakly stratified waves.

## Discussion

The quantified turbulent mixing of K_z_ = 10^−4^-10^−3^ m^2^ s^−1^ due to convection and IW near Texel-beach is one order of magnitude larger than found in the open ocean, while its overall mean, ballpark parameterization value, of 2×10^−5^ m^2^ s^−1^ equals typical open-ocean value [17], [18]. The associated overall mean <ε> = 6×10^−8^ W kg^−1^. This IW-mixing is two-three orders of magnitude smaller compared to SWW-breaking that is normally found at Texel-beach under average wind conditions, but it is three orders of magnitude larger than molecular diffusion. IW-turbulence is not driven by the atmosphere, as wind-stress and evaporative convection do not reach rapidly so deep when the water column warms on a summer-day. The beach observations resemble, in IW-groups, IW-asymmetry and KH-billows, much larger (10–40 m high) IW-breaking over deep-ocean large topography [15], [19]: this suggests beach-IW shoal coming in from the open sea possibly generated over the undulating sloping bottom; except that IW-beach-turbulence is smaller in magnitude. During another period with similar stratification, bands of different surface smoothness were visually observed at the surface coming in from the northwest at an estimated speed of 0.04 m s^−1^. Employing a two-layer internal wave model results in a phase speed of 0.03 m s^−1^ at given N, which is typical for internal waves in shallow waters (e.g., [20]). The moderate turbulence beach-values are likely due to absence of large scales found in the deep-ocean. The 1–2 m water depth limits overturns to an rms Ozmidov scale <L_O_> = 0.15 m, L_O_ = (ε/N^3^)^1/2^ ∼ d. This L_O_ is about 100 times smaller than found in 550 m over large topography, just like K_z_, roughly suggesting a linear dependence between K_z_ and L_O_. If so, we may parameterize N = K_z_^−1^ following d ∼ (K_z_/N)^1/2^ from (2), but we note variability in space and time can be very large, by several orders of magnitude.

From this beach experiment we learn that commonly rugged shallow seas like the southern North Sea can stratify during brief periods and are not well-mixed year-round, which is somewhat in contrast with predictions for seasonal stratification variations using bulk parameters [21]. This small-scale stratification hampers vertical turbulent mixing on the one hand but also supports motions in its interior, which generate sufficient vertical exchange, e.g., to maintain growth in the photic zone. The beach-site is useful for investigating SWW-IW-turbulence interaction and characteristics including fronts, convection and shear instabilities. Future experiments will include salinity and current measurements, and T-sensors mounted on several poles to obtain information on 3D propagation and evolution of IW-turbulence.

Using high-resolution T-sensors we have evidenced that the Italian mothers are right, of course, in sensing natural variability in the sea interior. Already in a cool sea such variations need only be 0.5–1°C to be sensed by humans. This T-difference provides stratification that is too weak to be a mechanical hazard to swimmers, who will lose only <5% of their energy to generate IW by their action [22]. They are likely more hampered by the shock of 1°C+ T-difference when naturally generated IW pass; that is, near a Texel-beach in early summer, as in the Adriatic cooler blobs may be felt more refreshing.
